# Bis(ethyl 2-amino-4-thia­zoleacetato-κ*N*)silver(I) nitrate

**DOI:** 10.1107/S1600536808032686

**Published:** 2008-10-18

**Authors:** Lai-Jun Zhang, Xing-Can Shen, Hong Liang

**Affiliations:** a Department of Chemistry, Shangrao Normal University, Shangrao 334001, People’s Republic of China; b Key Laboratory of Medicinal Chemical Resources and Molecular Engineering, College of Chemistry and Chemical Engineering, Guangxi Normal University, Guilin 541004, People’s Republic of China

## Abstract

In the title complex, [Ag(C_7_H_10_N_2_O_2_S)_2_]NO_3_, the Ag^I^ cation is bicoordinated in an almost linear configuration by two N-donor atoms of the thia­zole rings of two distinct ethyl 2-amino-4-thia­zoleacetate (EATA) ligands. The dihedral angle between the two thia­zole rings is 49.9°. A weak Ag⋯O (2.729 Å) inter­action between the Ag cation and one of the O atoms from the nitrate anion is observed, and a pseudo-dimer is formed through a weak Ag⋯S (3.490 Å) inter­action between the Ag cation and the S atom of the thia­zole ring of a symmetry-related mol­ecule. In the crystal structure, there are intra- and inter­molecular N—H⋯O hydrogen bonds. The occurrence of inter­molecular N—H⋯O hydrogen bonds results in the formation of two-dimensional sheets parallel to (010), which are further linked into a three-dimensional network through weak C—H⋯O inter­actions.

## Related literature

For related literature on the synthesis, see: Zhang *et al.* (2008 ▶). For related crystal structures, see: Dong *et al.* (2005 ▶); Fun *et al.* (2008 ▶); Lee & Lee (2007 ▶); Liu *et al.* (2007 ▶); Zhang *et al.* (2008 ▶). For related literature, see: Bolos *et al.* (1999 ▶); Chang *et al.* (1982 ▶); Garrison & Youngs (2005 ▶); Nomiya *et al.* (2000 ▶).
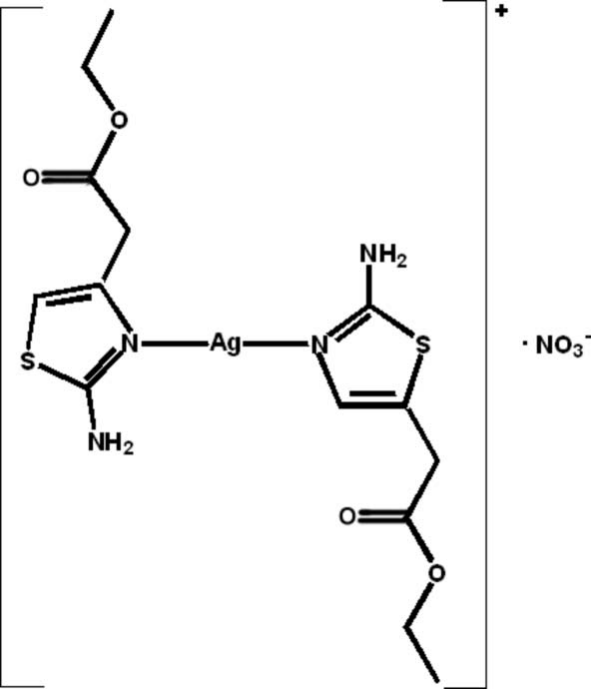


## Experimental

### 

#### Crystal data

[Ag(C_7_H_10_N_2_O_2_S)_2_]NO_3_
*M* *_r_* = 542.36
Triclinic, 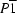

*a* = 7.4900 (15) Å
*b* = 12.350 (3) Å
*c* = 13.015 (3) Å
α = 109.32 (3)°
β = 101.83 (3)°
γ = 105.58 (3)°
*V* = 1035.6 (6) Å^3^
*Z* = 2
Mo *K*α radiation
μ = 1.22 mm^−1^
*T* = 293 (2) K
0.44 × 0.21 × 0.19 mm

#### Data collection

Bruker APEXII CCD area-detector diffractometer
Absorption correction: multi-scan (*SADABS*; Bruker, 2004 ▶) *T*_min_ = 0.616, *T*_max_ = 0.801
11916 measured reflections
3656 independent reflections
3518 reflections with *I* > 2σ(*I*)
*R*_int_ = 0.016

#### Refinement

*R*[*F* ^2^ > 2σ(*F* ^2^)] = 0.020
*wR*(*F* ^2^) = 0.053
*S* = 1.10
3656 reflections
265 parameters
H-atom parameters constrained
Δρ_max_ = 0.38 e Å^−3^
Δρ_min_ = −0.30 e Å^−3^

### 

Data collection: *APEX2* (Bruker, 2004 ▶); cell refinement: *APEX2*; data reduction: *APEX2*; program(s) used to solve structure: *SHELXS97* (Sheldrick, 2008 ▶); program(s) used to refine structure: *SHELXL97* (Sheldrick, 2008 ▶); molecular graphics: *ORTEPIII* (Burnett & Johnson, 1996 ▶), *ORTEP-3 for Windows* (Farrugia, 1997 ▶) and *CAMERON* (Pearce *et al.*, 2000 ▶); software used to prepare material for publication: *SHELXL97*.

## Supplementary Material

Crystal structure: contains datablocks I, global. DOI: 10.1107/S1600536808032686/dn2382sup1.cif

Structure factors: contains datablocks I. DOI: 10.1107/S1600536808032686/dn2382Isup2.hkl

Additional supplementary materials:  crystallographic information; 3D view; checkCIF report

## Figures and Tables

**Table 1:** Hydrogen-bond geometry (Å, °)

*D*—H⋯*A*	*D*—H	H⋯*A*	*D*⋯*A*	*D*—H⋯*A*
N3—H3*A*⋯O2^i^	0.86	2.13	2.955 (3)	162
N3—H3*B*⋯O6	0.86	2.16	3.019 (3)	175
N5—H5*A*⋯O1^ii^	0.86	2.04	2.886 (3)	169
N5—H5*B*⋯O2^iii^	0.86	2.14	2.972 (3)	161
C1—H1*C*⋯O3^iv^	0.96	2.53	3.298 (3)	137
C4—H4*A*⋯O3	0.97	2.60	3.492 (4)	153
C4—H4*B*⋯O2^iii^	0.97	2.43	3.330 (3)	155

Symmetry codes: (i) 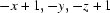; (ii) 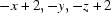; (iii) 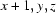; (iv) 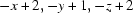.

**Fig. 1.**
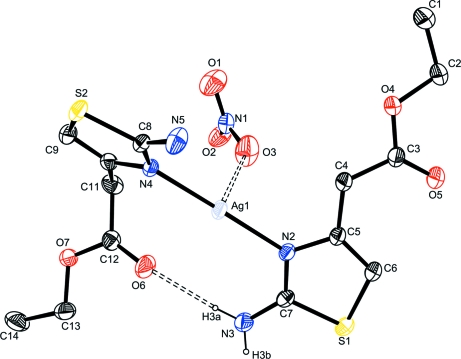
A view of complex (1), with the atom-labeling scheme. Displacement ellipsoids are drawn at the 30% probability level. H bond and weak Ag···O interactions are shown as dashed lines. Only H atoms involved in hydrogen bondings are shown. H atoms are drawn as small spheres of arbitrary radii.

**Fig. 2.**
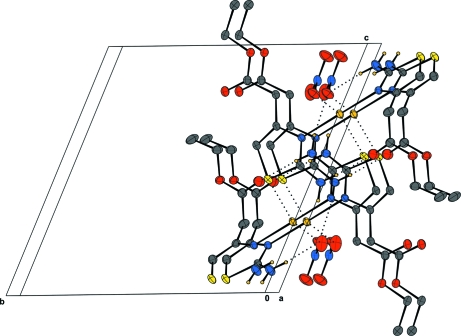
Partial packing view of (I), showing the formation of the pseudo dimer through weak Ag···S interactions and the two-dimensional network structure via intermolecular N—H···O hydrogen bonds. The weak interactions are represented as dashed lines. Hydrogen atoms not involved in hydrogen bonds were omitted for clarity.

## supplementary crystallographic information

### Comment

As a further extension of previous works on the synthesis of metal–organic complex containing pharmaceutical intermediates as ligands, here ethyl 2-amino-4-thiazoleacetate (EATA) is again applied as the ligand to obtain coordination complex with potential higher pharmacological activity (Bolos *et al.*, 1999; Chang *et al.*, 1982). Silver cation is chosen as central ion because many silver complexes show excellent antimicrobial effect (Garrison *et al.*, 2005; Nomiya *et al.*, 2000). Moreover, EATA has many conventional coordination atoms such N, O, S and many hydrogen atoms attached to N atoms, which may be in favor of the formation of diverse structures. In a recently similar research, we used cadmium chloride hydrate and 2-amino-4-thiazole acetic acid (ATAA) as starting materials to form a mononuclear compound, dichloridobis(2-amino-5-methyl-1,3-thiazole-*κN*)cadmium(II), due to the decarboxylization of ATAA under ethanol–water mixed-solvothermal reaction condition (Zhang *et al.*, 2008). To avoid potential instability of EATA under solvothermal condition, we carry out the reaction at low temperature as descried in experimental section and obtain an Ag-EATA complex as expected.

In the title complex, [Ag(C_7_H_10_N_2_O_2_S)_2_]NO_3_, the Ag^I^ cation is bicoordinated in an almost linear configuration by two N-donor atoms of thiazole rings of two distinct EATA ligand molecules (Fig. 1). Similar structures have been reported (Dong *et al.*, 2005; Fun *et al.*, 2008; Lee & Lee, 2007; Liu *et al.*, 2007). The N—Ag—N angle and dihedral angle between the two thiazole rings are respectively 175.88 (6) and 49.9°, and the average Ag—N distance is 2.138 (2) Å. In addition, there is a weak Ag···O interaction between silver cation and one of oxygen atoms from a nitrate group (Ag···O = 2.729 Å), while a pseudo dimer is built up through weak Ag···S [3.490 Å (Lee & Lee, 2007)] interaction between silver cation and one sulfur atom on a thiazole ring of a symmetry related molecule (Fig. 2). Thus, the title compound might also be regarded as a four-coordinated Ag complex with a N2OS donor set. In the crystal structure, due to Ag···O, Ag···S weak interactions and intermolecular N—H···O hydrogen bonds between adjacent molecules containing nitrate anions (Table 1), the molecules are extended to form two-dimensional layers (Fig. 2) parallel to the (010) plane, which are further linked to a three dimensional network through weak C—H···O interactions (Table 1).

### Experimental

To 10 ml ethanol solution containing ethyl 2-amino-4-thiazoleacetate (EATA) (0.186 g, 1 mmol), AgNO_3_ (0.170 g, 1 mmol) was added and the resulting mixture was stirred in the dark at room temperature for 4 h. After the filtrate had been allowed to stand overnight at a refrigerator temperature of 4 °C, the colourless block single crystals suitable for X-ray diffraction were obtained. Yield: 45.3% (based on Ag).

### Refinement

All H atoms attached to C or N atoms were placed in geometrically (C—H = 0.93–0.97 Å, N—H = 0.86 Å) and refined using a riding model, with *U*_iso_(H) = 1.2 or 1.5 *U*_eq_(*C*) and *U*_iso_(H) = 1.2 *U*_eq_(*N*).

### Crystal data

**Table:**

[Ag(C_7_H_10_N_2_O_2_S)_2_]NO_3_	*Z* = 2
*M**_r_* = 542.36	*F*(000) = 548
Triclinic, *P*1	*D*_x_ = 1.739 Mg m^−^^3^
Hall symbol: -P 1	Mo *K*α radiation, λ = 0.71073 Å
*a* = 7.4900 (15) Å	Cell parameters from 3656 reflections
*b* = 12.350 (3) Å	θ = 1.8–25.1°
*c* = 13.015 (3) Å	µ = 1.22 mm^−^^1^
α = 109.32 (3)°	*T* = 293 K
β = 101.83 (3)°	Block, colourless
γ = 105.58 (3)°	0.44 × 0.21 × 0.19 mm
*V* = 1035.6 (6) Å^3^	

### Data collection

**Table:**

Bruker APEXII CCD area-detector diffractometer	3656 independent reflections
Radiation source: fine-focus sealed tube	3518 reflections with *I* > 2σ(*I*)
graphite	*R*_int_ = 0.016
φ and ω scans	θ_max_ = 25.1°, θ_min_ = 1.8°
Absorption correction: multi-scan (SADABS; Bruker, 2004)	*h* = −8→8
*T*_min_ = 0.616, *T*_max_ = 0.801	*k* = −14→14
11916 measured reflections	*l* = −15→15

### Refinement

**Table:**

Refinement on *F*^2^	Secondary atom site location: difference Fourier map
Least-squares matrix: full	Hydrogen site location: inferred from neighbouring sites
*R*[*F*^2^ > 2σ(*F*^2^)] = 0.020	H-atom parameters constrained
*wR*(*F*^2^) = 0.053	*w* = 1/[σ^2^(*F*_o_^2^) + (0.0247*P*)^2^ + 0.5391*P*] where *P* = (*F*_o_^2^ + 2*F*_c_^2^)/3
*S* = 1.10	(Δ/σ)_max_ = 0.001
3656 reflections	Δρ_max_ = 0.38 e Å^−^^3^
265 parameters	Δρ_min_ = −0.30 e Å^−^^3^
0 restraints	Extinction correction: SHELXL97 (Sheldrick, 2008), Fc^*^=kFc[1+0.001xFc^2^λ^3^/sin(2θ)]^-1/4^
0 constraints	Extinction coefficient: 0.0267 (10)
Primary atom site location: structure-invariant direct methods	

### Special details

**Table:**

Experimental. FT–IR (KBr, cm^-1^):3409 vs, 3296 ms, 3206 ms, 3152 s, 2987 m, 2942 w, 2906 w, 2733 w, 2346 w, 1740 vs, 1706 vs, 1627 vs, 1565 m, 1541 vs, 1526 ms, 1477 m, 1448 m, 1402 ms, 1384 vs, 1321 s, 1249 ms, 1174 vs, 1131 ms, 1115 ms, 1029 ms, 995 w, 980 m, 948 w, 826 w, 752 w, 717 m, 658 w, 596 w, 547 w.
Geometry. All e.s.d.'s (except the e.s.d. in the dihedral angle between two l.s. planes) are estimated using the full covariance matrix. The cell e.s.d.'s are taken into account individually in the estimation of e.s.d.'s in distances, angles and torsion angles; correlations between e.s.d.'s in cell parameters are only used when they are defined by crystal symmetry. An approximate (isotropic) treatment of cell e.s.d.'s is used for estimating e.s.d.'s involving l.s. planes.
Refinement. Refinement of *F*^2^ against ALL reflections. The weighted *R*-factor *wR* and goodness of fit *S* are based on *F*^2^, conventional *R*-factors *R* are based on *F*, with *F* set to zero for negative *F*^2^. The threshold expression of *F*^2^ > σ(*F*^2^) is used only for calculating *R*-factors(gt) *etc*. and is not relevant to the choice of reflections for refinement. *R*-factors based on *F*^2^ are statistically about twice as large as those based on *F*, and *R*-factors based on ALL data will be even larger.

### Fractional atomic coordinates and isotropic or equivalent isotropic displacement parameters (Å^2^)

**Table:**

	*x*	*y*	*z*	*U*_iso_*/*U*_eq_	
Ag1	0.85033 (2)	−0.013259 (13)	0.716062 (13)	0.04021 (8)	
S1	0.91350 (9)	0.16283 (5)	0.45565 (5)	0.04745 (15)	
S2	0.90581 (10)	−0.23263 (6)	0.94281 (5)	0.05325 (16)	
O6	0.5425 (3)	−0.22530 (17)	0.55153 (16)	0.0687 (5)	
O7	0.3231 (2)	−0.39127 (14)	0.54375 (13)	0.0474 (4)	
O5	1.2202 (3)	0.48839 (16)	0.81321 (16)	0.0736 (6)	
O4	1.2230 (3)	0.45176 (13)	0.96884 (13)	0.0515 (4)	
N3	0.6973 (3)	−0.05628 (17)	0.44144 (16)	0.0516 (5)	
H3B	0.6555	−0.1077	0.4702	0.062*	
H3A	0.6574	−0.0782	0.3682	0.062*	
N2	0.8931 (2)	0.09958 (15)	0.62234 (14)	0.0345 (3)	
N5	1.1602 (3)	−0.06100 (19)	0.90954 (18)	0.0560 (5)	
H5B	1.1924	−0.0101	0.8787	0.067*	
H5A	1.2491	−0.0674	0.9583	0.067*	
N4	0.8272 (2)	−0.12730 (14)	0.80909 (14)	0.0344 (3)	
C8	0.9742 (3)	−0.12943 (18)	0.88205 (17)	0.0374 (4)	
C9	0.6656 (4)	−0.2735 (2)	0.8643 (2)	0.0510 (6)	
H9	0.5601	−0.3322	0.8663	0.061*	
C10	0.6507 (3)	−0.20965 (18)	0.79984 (17)	0.0390 (5)	
C11	0.4644 (3)	−0.2204 (2)	0.7219 (2)	0.0510 (6)	
H11B	0.4572	−0.1392	0.7404	0.061*	
H11A	0.3544	−0.2708	0.7346	0.061*	
C12	0.4492 (3)	−0.2767 (2)	0.59729 (19)	0.0445 (5)	
C13	0.2915 (4)	−0.4515 (2)	0.42144 (19)	0.0558 (6)	
H13B	0.4116	−0.4592	0.4089	0.067*	
H13A	0.2505	−0.4037	0.3820	0.067*	
C7	0.8230 (3)	0.05670 (19)	0.50993 (17)	0.0368 (4)	
C6	1.0455 (4)	0.2706 (2)	0.59426 (19)	0.0453 (5)	
H6	1.1254	0.3511	0.6134	0.054*	
C5	1.0172 (3)	0.22243 (18)	0.67040 (17)	0.0360 (4)	
C4	1.0999 (3)	0.27987 (18)	0.79796 (18)	0.0428 (5)	
H4B	1.2009	0.2488	0.8200	0.051*	
H4A	0.9969	0.2515	0.8280	0.051*	
C3	1.1861 (3)	0.41749 (19)	0.85648 (19)	0.0420 (5)	
C2	1.2986 (4)	0.5826 (2)	1.0409 (2)	0.0542 (6)	
H2B	1.2073	0.6200	1.0187	0.065*	
H2A	1.4229	0.6228	1.0335	0.065*	
C1	1.3247 (4)	0.5950 (2)	1.1613 (2)	0.0621 (7)	
H1C	1.3663	0.6804	1.2117	0.093*	
H1B	1.4220	0.5627	1.1838	0.093*	
H1A	1.2027	0.5500	1.1661	0.093*	
C14	0.1377 (5)	−0.5744 (3)	0.3773 (3)	0.0772 (9)	
H14C	0.0217	−0.5659	0.3937	0.116*	
H14B	0.1828	−0.6226	0.4138	0.116*	
H14A	0.1085	−0.6149	0.2957	0.116*	
O3	0.6428 (4)	0.1229 (2)	0.8055 (2)	0.0928 (8)	
O2	0.3485 (3)	0.09345 (19)	0.80013 (16)	0.0650 (5)	
O1	0.5549 (3)	0.1152 (2)	0.9498 (2)	0.0903 (7)	
N1	0.5176 (3)	0.10894 (16)	0.85172 (17)	0.0447 (4)	

### Atomic displacement parameters (Å^2^)

**Table:**

	*U*^11^	*U*^22^	*U*^33^	*U*^12^	*U*^13^	*U*^23^
Ag1	0.04525 (12)	0.03583 (10)	0.03859 (11)	0.00714 (7)	0.00920 (7)	0.02277 (7)
S1	0.0633 (4)	0.0491 (3)	0.0388 (3)	0.0198 (3)	0.0174 (3)	0.0284 (2)
S2	0.0699 (4)	0.0490 (3)	0.0467 (3)	0.0141 (3)	0.0173 (3)	0.0335 (3)
O6	0.0612 (11)	0.0628 (11)	0.0586 (11)	−0.0110 (9)	0.0136 (9)	0.0270 (9)
O7	0.0490 (9)	0.0414 (8)	0.0380 (8)	0.0015 (7)	0.0134 (7)	0.0123 (6)
O5	0.1150 (17)	0.0405 (9)	0.0550 (10)	0.0061 (10)	0.0262 (11)	0.0260 (8)
O4	0.0712 (11)	0.0317 (7)	0.0418 (8)	0.0070 (7)	0.0152 (8)	0.0144 (6)
N3	0.0577 (12)	0.0486 (11)	0.0350 (9)	0.0045 (9)	0.0063 (8)	0.0182 (8)
N2	0.0366 (9)	0.0355 (8)	0.0344 (8)	0.0114 (7)	0.0120 (7)	0.0189 (7)
N5	0.0381 (10)	0.0671 (13)	0.0624 (13)	0.0072 (9)	0.0044 (9)	0.0426 (11)
N4	0.0361 (9)	0.0317 (8)	0.0326 (8)	0.0062 (7)	0.0098 (7)	0.0157 (7)
C8	0.0441 (11)	0.0355 (10)	0.0329 (10)	0.0092 (9)	0.0120 (8)	0.0187 (8)
C9	0.0569 (14)	0.0420 (12)	0.0480 (13)	0.0014 (10)	0.0224 (11)	0.0210 (10)
C10	0.0403 (11)	0.0323 (10)	0.0351 (10)	0.0033 (8)	0.0144 (8)	0.0093 (8)
C11	0.0375 (12)	0.0499 (13)	0.0487 (13)	0.0064 (10)	0.0122 (10)	0.0086 (10)
C12	0.0336 (11)	0.0438 (11)	0.0466 (12)	0.0054 (9)	0.0075 (9)	0.0176 (10)
C13	0.0608 (15)	0.0563 (14)	0.0383 (12)	0.0090 (12)	0.0167 (11)	0.0147 (11)
C7	0.0384 (10)	0.0431 (11)	0.0361 (10)	0.0169 (9)	0.0134 (8)	0.0222 (9)
C6	0.0579 (13)	0.0384 (11)	0.0436 (12)	0.0133 (10)	0.0185 (10)	0.0236 (9)
C5	0.0397 (11)	0.0347 (10)	0.0397 (11)	0.0137 (8)	0.0150 (9)	0.0209 (8)
C4	0.0528 (13)	0.0345 (10)	0.0387 (11)	0.0094 (9)	0.0119 (9)	0.0191 (9)
C3	0.0427 (11)	0.0375 (11)	0.0455 (12)	0.0098 (9)	0.0148 (9)	0.0202 (9)
C2	0.0675 (16)	0.0313 (11)	0.0519 (13)	0.0083 (10)	0.0149 (12)	0.0133 (10)
C1	0.0799 (19)	0.0441 (13)	0.0509 (14)	0.0155 (12)	0.0170 (13)	0.0141 (11)
C14	0.082 (2)	0.0566 (16)	0.0553 (16)	−0.0010 (14)	0.0250 (15)	−0.0027 (13)
O3	0.0874 (16)	0.0820 (15)	0.147 (2)	0.0438 (13)	0.0819 (16)	0.0554 (15)
O2	0.0496 (10)	0.0931 (14)	0.0609 (11)	0.0222 (9)	0.0117 (8)	0.0483 (10)
O1	0.0676 (13)	0.1224 (19)	0.0748 (14)	0.0182 (13)	−0.0027 (11)	0.0614 (14)
N1	0.0436 (10)	0.0380 (9)	0.0576 (12)	0.0144 (8)	0.0184 (9)	0.0250 (8)

### Geometric parameters (Å, °)

**Table:**

Ag1—N4	2.1361 (17)	C11—C12	1.504 (3)
Ag1—N2	2.1396 (17)	C11—H11B	0.9700
S1—C6	1.725 (3)	C11—H11A	0.9700
S1—C7	1.734 (2)	C13—C14	1.474 (4)
S2—C9	1.719 (3)	C13—H13B	0.9700
S2—C8	1.730 (2)	C13—H13A	0.9700
O6—C12	1.195 (3)	C6—C5	1.338 (3)
O7—C12	1.319 (3)	C6—H6	0.9300
O7—C13	1.453 (3)	C5—C4	1.485 (3)
O5—C3	1.189 (3)	C4—C3	1.496 (3)
O4—C3	1.325 (3)	C4—H4B	0.9700
O4—C2	1.449 (3)	C4—H4A	0.9700
N3—C7	1.325 (3)	C2—C1	1.489 (3)
N3—H3B	0.8600	C2—H2B	0.9700
N3—H3A	0.8600	C2—H2A	0.9700
N2—C7	1.311 (3)	C1—H1C	0.9600
N2—C5	1.390 (3)	C1—H1B	0.9600
N5—C8	1.321 (3)	C1—H1A	0.9600
N5—H5B	0.8600	C14—H14C	0.9600
N5—H5A	0.8600	C14—H14B	0.9600
N4—C8	1.311 (3)	C14—H14A	0.9600
N4—C10	1.389 (3)	O3—N1	1.218 (3)
C9—C10	1.336 (3)	O2—N1	1.236 (3)
C9—H9	0.9300	O1—N1	1.221 (3)
C10—C11	1.491 (3)		
			
N4—Ag1—N2	175.88 (6)	H13B—C13—H13A	108.5
C6—S1—C7	89.44 (10)	N2—C7—N3	125.01 (19)
C9—S2—C8	89.17 (11)	N2—C7—S1	113.38 (16)
C12—O7—C13	116.29 (18)	N3—C7—S1	121.60 (16)
C3—O4—C2	117.66 (18)	C5—C6—S1	110.73 (17)
C7—N3—H3B	120.0	C5—C6—H6	124.6
C7—N3—H3A	120.0	S1—C6—H6	124.6
H3B—N3—H3A	120.0	C6—C5—N2	114.81 (19)
C7—N2—C5	111.58 (17)	C6—C5—C4	129.80 (19)
C7—N2—Ag1	123.44 (14)	N2—C5—C4	115.38 (17)
C5—N2—Ag1	124.52 (13)	C5—C4—C3	117.78 (18)
C8—N5—H5B	120.0	C5—C4—H4B	107.9
C8—N5—H5A	120.0	C3—C4—H4B	107.9
H5B—N5—H5A	120.0	C5—C4—H4A	107.9
C8—N4—C10	110.89 (17)	C3—C4—H4A	107.9
C8—N4—Ag1	125.47 (13)	H4B—C4—H4A	107.2
C10—N4—Ag1	123.65 (14)	O5—C3—O4	123.3 (2)
N4—C8—N5	125.14 (19)	O5—C3—C4	127.6 (2)
N4—C8—S2	113.98 (15)	O4—C3—C4	109.03 (18)
N5—C8—S2	120.88 (17)	O4—C2—C1	106.58 (19)
C10—C9—S2	110.93 (17)	O4—C2—H2B	110.4
C10—C9—H9	124.5	C1—C2—H2B	110.4
S2—C9—H9	124.5	O4—C2—H2A	110.4
C9—C10—N4	115.0 (2)	C1—C2—H2A	110.4
C9—C10—C11	125.5 (2)	H2B—C2—H2A	108.6
N4—C10—C11	119.43 (19)	C2—C1—H1C	109.5
C10—C11—C12	112.00 (19)	C2—C1—H1B	109.5
C10—C11—H11B	109.2	H1C—C1—H1B	109.5
C12—C11—H11B	109.2	C2—C1—H1A	109.5
C10—C11—H11A	109.2	H1C—C1—H1A	109.5
C12—C11—H11A	109.2	H1B—C1—H1A	109.5
H11B—C11—H11A	107.9	C13—C14—H14C	109.5
O6—C12—O7	123.2 (2)	C13—C14—H14B	109.5
O6—C12—C11	124.5 (2)	H14C—C14—H14B	109.5
O7—C12—C11	112.28 (19)	C13—C14—H14A	109.5
O7—C13—C14	107.5 (2)	H14C—C14—H14A	109.5
O7—C13—H13B	110.2	H14B—C14—H14A	109.5
C14—C13—H13B	110.2	O3—N1—O1	122.3 (2)
O7—C13—H13A	110.2	O3—N1—O2	119.3 (2)
C14—C13—H13A	110.2	O1—N1—O2	118.4 (2)

### Hydrogen-bond geometry (Å, °)

**Table:**

*D*—H···*A*	*D*—H	H···*A*	*D*···*A*	*D*—H···*A*
N3—H3A···O2^i^	0.86	2.13	2.955 (3)	162
N3—H3B···O6	0.86	2.16	3.019 (3)	175
N5—H5A···O1^ii^	0.86	2.04	2.886 (3)	169
N5—H5B···O2^iii^	0.86	2.14	2.972 (3)	161
C1—H1C···O3^iv^	0.96	2.53	3.298 (3)	137
C4—H4A···O3	0.97	2.60	3.492 (4)	153
C4—H4B···O2^iii^	0.97	2.43	3.330 (3)	155

Symmetry codes: (i) −*x*+1, −*y*, −*z*+1; (ii) −*x*+2, −*y*, −*z*+2; (iii) *x*+1, *y*, *z*; (iv) −*x*+2, −*y*+1, −*z*+2.
